# Supplementary material for: Repeated Intravenous Administration of Human Neural Stem Cells Producing Choline Acetyltransferase Exerts Anti-Aging Effects in Male F344 Rats
Source: Cells. 2023 Nov 26;12(23):2711. doi: 10.3390/cells12232711 (PMC10706332; doi:10.3390/cells12232711)
Supplement: Supplementary file 1 [file cells-12-02711-s001.zip › cells-2625664-supplementary.pdf]

Supplementary Table S1. Primer sequences used for the analysis of cholinergic and dopaminergic system markers in the brain tissue

**Gene**	**Primer**	**Sequences**
ChAT	Forward	5′-TTCTTGTTGCTTGTCATCATATGTTTC-3′
	Reverse	5′-CGGTGTTGGTGTGTGAGCATT-3′
VAChT	Forward	5′-CGCGCTCACCACTTGTAACAT-3′
	Reverse	5′-CGGCCATTGTGTGCTTCA-3′
ChT1	Forward	5′-CCCATGCGCTTTCCATAGAT-3′
	Reverse	5′-AGGTGGCCTGTTTTTTGCAA-3′
m1AChR	Forward	5′-CCTCCCAAAAGCTCCCCA-3′
	Reverse	5′-TGTCCCGGAAGGCTTTGT-3′
nAChR α5	Forward	5′-CAACATCCACCACCGCTCTT-3′
	Reverse	5′-GCAGCTTGGGAAGCTTGTG-3′
nAChR β2	Forward	5′-CTTCTATTCCAATGCTGTGGTCTCCTATG-3′
	Reverse	5′-AGCGGTACGTCGAGGGAGGTG-3′
AChE	Forward	5′-CCTGTGCGGGCAAAATTG-3′
	Reverse	5′-CCTGGATCCCTCGCTGAA-3′
TH	Forward	5′-CCTCCTTGTCTCGGGCTGTAA-3′
	Reverse	5′-CTGAGCTTGTCCTTGGCGTCA-3′
VMAT2	Forward	5′-TTAGGAATTTACAACTCGTCA-3′
	Reverse	5′-GTGAAACTCATTTCTACATTG-3′
DAT	Forward	5′-GGACCAATGTCTTCAGTGGTGGC-3′
	Reverse	5′-GGATCCATGGGAGGTCCATGG-3′
D1R	Forward	5′-CGCGGATCCACAAGCTTCTACAGGATTGCCCAGAAGCAA-3′
	Reverse	5′-CGCGGATCCGAAGCTTTCACTTTAGAACTTTCGTCTCCCT-3′
D2R	Forward	5′-CCTTCATCGTCACTCTGCTGG-3′
	Reverse	5′-CTCCATTTCCAGCTCCTGAG-3′
CCL11	Forward	5′-AGATGCACGCTGAAAGCCATAGTC-3′
	Reverse	5′-GGTGCCGATATTCTCCCATAGCAT-3′
GAPDH	Forward	5′-AACGGATTTGGCCGTATCGG-3′
	Reverse	5′-AGCCTTCTCCATGGTGGTGAAGAC-3′

ChAT: choline acetyltransferase, VAChT: vesicular acetylcholine transporter, ChT1: choline transporter 1, m1-AChR: muscarinic 1 acetylcholine receptor, nAChR, nicotinic acetylcholine receptor, AChE: acetylcholinesterase, TH: tyrosine hydroxylase, VMAT2: vesicular monoamine transporter 2, DAT: dopamine transporter, DR: dopamine receptor, CCL11: chemokine (C-C motif) ligand 11.
